# Diabetes pay-for-performance program can reduce all-cause mortality in patients with newly diagnosed type 2 diabetes mellitus

**DOI:** 10.1097/MD.0000000000019139

**Published:** 2020-02-14

**Authors:** Fang-Ping Kung, Ching-Fang Tsai, Chin-Li Lu, Li-Chung Huang, Chieh-Hsiang Lu

**Affiliations:** a Division of Endocrinology and Metabolism, Department of Internal Medicine, Ditmanson Chia-Yi Christian Hospital; b Department of Medical Research, Ditmanson Chia-Yi Christian Hospital, Chia-Yi City; c Graduate Institute of Food Safety, College of Agriculture and Natural Resources, National Chung-Hsing University, Taichung; d Division of Psychiatry, Ditmanson Chia-Yi Christian Hospital, Chia-Yi City; e Kaohsiung Christian Hospital, Kaohsiung City, Taiwan.

**Keywords:** all-cause mortality, diabetes mellitus, pay-for-performance program

## Abstract

This study aimed to examine the effect of a diabetes pay-for-performance (P4P) program on all-cause mortality in patients with newly diagnosed type 2 diabetes mellitus. Using a Taiwanese representative nationwide cohort, we recruited 5478 patients with newly diagnosed type 2 diabetes enrolled in the P4P program within 5 years after a diagnosis of diabetes between January 1, 2002 and December 31, 2010 and individuals not enrolled in the P4P program were recruited as the control group matched 1:1 with the study group. We used multivariate Cox proportional hazard models analysis to investigate the effect of the P4P program and adherence on all-cause mortality. A total of 250 patients died in the P4P group compared to 395 in the control group (mortality rate 104 vs 169 per 10,000 person-years, respectively, *P* < .0001). The control group also had more comorbidities. Patients enrolled in the P4P program demonstrated significant long-term survival benefits, of which the adjusted hazard ratio (aHR) for all-cause mortality was 0.58 [95% CI (0.48–0.69)]. In the study group, better adherence to the P4P program resulted in a greater reduction in mortality, with aHRs [95% CI] of 0.48 [0.38–0.62] and 0.36 [0.26–0.49] in subjects with a minimum 1-year and 2-year good P4P adherence, respectively. Participating in the P4P program within 5 years after the diagnosis of diabetes resulted in a significant reduction in all-cause mortality, and this effect was particularly pronounced in the patients with better adherence to the P4P program.

## Abstract 2

Supplemental Digital Content is available in the text

## 1 Introduction

Diabetes mellitus is a growing healthcare problem worldwide, and is associated with increased microvascular and macrovascular complications. About 40% of US adults are at an increased risk of developing diabetes mellitus in their lifetime.^[1]^ According to World Health Organization estimates, the number of people with diabetes will increase from 177 million people in 2000 to 300 million by 2025.^[2]^ To improve the quality of diabetes care, many countries including the USA, UK, Australia and Germany have launched diabetes pay-for-performance (P4P) programs, which are healthcare payment models where financial incentives are given for achieving pre-set health outcome targets.

In 2001, the Bureau of National Health Insurance (NHI) in Taiwan implemented a P4P program, with the aim of improving the quality of diabetes care, provided by physicians, certified diabetes educators and registered dietitians. This program focuses on patient-centered medical care, diabetes self-management education, improved adherence to diabetes guidelines and screening for diabetes-related complications in order to reduce chronic vascular complications, national health expenditure and long-term mortality.

Previous studies have reported that patients enrolled in P4P programs had better adherence to the guideline-recommended examinations,^[3,4]^ had better clinical processes of care (e.g. HbA1c) and intermediate outcomes,^[5,6]^ decreased diabetes-related hospitalizations and inpatient costs,^[7]^ but increased rates of severe hypoglycemia requiring emergency medical care^[8]^ and increased outpatient expenses due to more regular follow-up visits.^[4]^ Patients with type 2 diabetes are associated with a two-fold increase in mortality^[9,10]^ and a reduction in life expectancy by about 6 years compared with individuals without diabetes. In addition, the 10-year follow-up UKPDS study demonstrated a relative risk reduction of 13% in all-cause mortality^[11]^ with timely multidisciplinary team care to treat diabetic patients earlier in the course of the disease. Previous studies in Taiwan has shown the potential benefit of diabetes P4P programs in reducing risk of all-cause mortality among type 2 diabetic patients,^[12,13]^ however, the enrolled subjects may or may not be newly diagnosed type 2 diabetes mellitus and no specific time interval of enrollment of P4P program after the diagnosis of type 2 diabetes mellitus. Therefore, we conducted this cohort study of patients with newly diagnosed type 2 diabetes mellitus who participated early in a P4P program to investigate its effect and adherence of P4P program on mortality compared with matched diabetic patients who received standard medical care.

## 2 Methods

### 2.1 Sources of data and study population

Data were collected from the reimbursement records of the NHI program stored in the NHI Research Database (NHIRD) including complete personal claims data which are provided for research purposes. The NHI program was instituted in 1995 and now covers 99% of the population in Taiwan. In this study, we analyzed a nationwide representative cohort of 1 million people who were randomly sampled from the more than 23 million insured beneficiaries registered in the NHIRD. The inpatient and outpatient medical claims data and all personal information for this cohort of 1 million beneficiaries were extracted from 1996 to 2011. We recruited patients with newly diagnosed type 2 diabetes enrolled in the P4P program within 5 years after the diagnosis of diabetes between January 1, 2002 and December 31, 2010. The study was conducted after obtaining ethical approval of the Institutional Review Board of Chiayi Christian Hospital (approval no. CYCH-IRB-103046).

### 2.2 Study design

Patients were defined as having diabetes mellitus if they had at least 1 hospital admission with a diagnostic code of diabetes mellitus (ICD-9-CM code 250) or 3 or more outpatient visits with the same ICD-9-CM code within a 1-year period. The date of diabetes onset was recorded as the date of the first visit for diabetes. To define newly diagnosed cases of type 2 diabetes since 2002, patients with medical records listing diabetes before December 31, 2001 were excluded. Patients with type 1 diabetes and those under the age of 18 years were also excluded from this study. Patients were allocated to the P4P group if they had newly diagnosed type 2 diabetes between January 1, 2002 and December 31, 2010 and were enrolled in the P4P program (internal code in the NHI system: P14xx) within 5 years after the initial diagnosis of diabetes. The control group was randomly sampled at a 1-to-1 ratio to the P4P group, and included those who fulfilled the inclusion criteria listed above during the same time period, but who had never been enrolled in a P4P program. In addition, inclusion criteria for the patients in the control group were being alive on the index date of the P4P patient and both group not having any hospital admissions during the 1-year period before the index date to reduce the effect of medically fragile patients with other underlying disease on mortality. The controls were matched to the P4P group by gender, ±5 years of age, and ±3 months of the date of diabetes onset. The index date was defined as the date of first enrollment in the P4P program as evidenced by the presence of a specific code (internal code in the NHI system: P1407) in the claims data. Each matched pair were followed from the index date until death, the end of the study (December 31, 2011), or the date of withdrawal from the NHI program. The date of death was defined as that when the beneficiaries withdrew from the NHI program due to death.

### 2.3 Definition of comorbidities and other variables

Major comorbidities were defined as those with at least 1 admission record or at least 2 outpatient visits for a certain diagnosis within 3 years before the index date. The comorbidities included hypertension (ICD-9 401–402 and 405), hyperlipidemia (ICD-9 272.0–272.4), coronary artery disease (ICD-9 414.8 and 414.9), peripheral vascular disease (ICD-9 440–443, 447, and 557), stroke/cerebrovascular disease (ICD-9 430–438), heart failure (ICD-9 398.91, 402.01 402.11, 402.91,404.01, 404.03, 404.11, 404.13, 404.91,404.93, and 428–428.9), liver disease (ICD-9 570, 571, and 572.4), renal disease (ICD-9 016.0, 095.4, 189.0, 189.9, 223.0, 236.91,250.4,271.4, 274.1, 283.11, 403.X1,404.X2, 404.X3, 440.1, 442.1, 447.3, 572.4, 580–588, 591, 642.1,646.2, 753.12–753.17,753.19,753.2, and 794.4), chronic obstructive pulmonary disease (ICD-9 491–494,496 and 510), rheumatoid arthritis/collagen deficiency disease (ICD-9 701.0, 710.0–710.9, 714.0–714.9,720.0–720.9, and 725), gastrointestinal bleeding (ICD-9 456.0–456.2, 530.7, 531–534, 569.84, 569.85, and 578), adrenal disorders (ICD-9 255), hyperthyroidism (ICD-9 242), hypothyroidism (ICD-9 243–244.2, 244.8, and 244.9), psychoses (ICD-9 295.00–298.9), depression (ICD-9 300.4, 301.12, 309.1, and 311), dementia (ICD-9 290, 296.2x, 296.3x, 291.1, 29.2, and 294) and cancer (ICD-9 140–239). Several studies have been performed to validate the diagnostic accuracy in the National Health Insurance Research Database in Taiwan.^[14–19]^ All of these study results confirmed the accuracy of most of the comorbidities included in our study are high, which suggested the minimal estimation bias induced by misclassification in our study. Other variables including anti-diabetic agents, hospital level, and region of hospital location and frequency of outpatient visits were adjusted in the analysis. Exposure to anti-diabetic agents was defined according the drug prescriptions recorded in the 6 months prior to death or the end of the study, and the anti-diabetic agents were categorized as: metformin only, sulfonylurea only, insulin only, metformin + sulfonylurea, metformin + insulin, sulfonylurea + insulin, metformin + sulfonylurea + insulin, and others. The hospital level was defined as a medical center, regional hospital, district hospital, and primary clinic. The hospital location was defined as Taipei + Northern region, central region, southern + Kao-ping region, and eastern region according to the geographic regions of Taiwan. The frequency of outpatient visits was the average annual number of outpatient visits, calculated from the index date until death. The duration of diabetes mellitus was calculated from the date of a first visit for diabetes until the end of follow-up.

### 2.4 *The* workflow of the P4P program in Taiwan

The P4P program conducted in Taiwan is a system of multidisciplinary team care whose members included physicians, registered nurses, dietitian, and pharmacists. Only physicians who are certified by Taiwanese Association of Diabetes Educators (TADE) can enroll diabetic patients in the P4P program. In the setting of the P4P program, an enrollee of the P4P program is advised to visit the physician once every 3 months to complete a structured care, which is defined in the initial enrollment visit (internal code P1407), continuing care visits (internal code P1408) and an annual evaluation visit (internal code P1409), respectively. The components of the structured care included medical history, physical examination, laboratory evaluation, evaluation of management plan, and diabetes self-management education. This program is a reward-based, not a penalty-based system. In addition to regular physician fees, physicians can get extra incentive payments from the P4P program. In order to claim the P4P reimbursement, data of the “must-do” laboratory tests and examinations, which is composed of blood sugar, HbA1C, LDL, triglyceride, serum creatinine, urine albumin/creatinine ratio, systolic and diastolic blood pressure, eye fundus examination, and foot examination for initial enrollment visit and annual evaluation visit, and include blood sugar, HbA1C, systolic and diastolic blood pressure for continuing care visit, must be uploaded to Bureau of Health Promotion. An annual composite outcome score, which is calculated by the adherence rate of outpatient visit, the proportion of enrollees with HbA1C < 7.0%, the proportion of enrollees with HbA1C >9%, the proportion of enrollees with LDL < 100 mg/dl and the proportion of enrollees with LDL >130 mg/dl, are used to assess physicians annual performance. Only the top 25% best performing physicians will be rewarded. Therefore, physicians have more incentives to improve quality of diabetes care to get the bonus payments from the P4P program.

The influence of adherence to the P4P program on mortality was also of interest in this study. In the year following the initial visit (internal code P1407), there should have been 2 comprehensive follow-up visits (internal code P1408) and an annual evaluation visit (internal code P1409) for the P4P program every 3 months. The enrolled patients may not have visited regularly or dropped out early, and good adherence was defined as having out-patient department visits for the P4P program at least twice a year, otherwise adherence was defined as being poor.

### 2.5 Statistical methods

To compare the characteristics between the P4P and control groups, we used the *t* test to compare continuous variables and the chi-square test to compare categorized variables. The independent variables included in the multivariate Cox proportional hazards model with robust sandwich variance are age, outpatient visits, antidiabetic agents, hospital level, geographical region, and comorbidities (hypertension, hyperlipidemia, coronary artery disease, peripheral vascular disease, cerebrovascular disease, heart failure, liver disease, renal disease, chronic obstructive pulmonary disease, rheumatoid arthritis/collagen deficiency disease, gastrointestinal bleeding, adrenal disorder, hyperthyroidism, hypothyroidism, psychoses, depression, dementia and cancer) and we used adjusted hazard ratios (aHRs) and their 95% confidence intervals (CIs) to investigate the effect of P4P program on all-cause mortality. To further examine whether the effect of the P4P program on mortality differed according to the duration of adherence to the program, we additionally performed 2 subgroup analyses in which the diabetic patients with good adherence for at least 1 year or at least 2 years were compared with their matched controls in the conventional Cox regression models. The reason why we did not choose to evaluate the interrelationship between adherence status and mortality by treating adherence status and comorbidities as time-dependent variables in Cox regression models is because of the difficulty to clarify the true causal relationship between the adherence status and mortality and the possible caveat to adjust for sequelae.^[20,21]^ Furthermore, we also evaluate whether the beneficial effect of P4P program is consistent between groups stratified by age, insulin use and other comorbidities. The significantly different effects of the P4P program in the various subgroups were presented by aHRs with 95% CIs and depicted in a forest plot. The Kaplan–Meier method was used to draw survival curves which were then compared by the log-rank test (Supplemental Figure). All statistical analyses were conducted using SAS software version 9.3 (SAS Institute, Cary, NC, USA.). A two-tailed *P*-value of less than .05 was considered to be statistically significant.

## 3 Results

### 3.1 Baseline characteristics and comorbidities of the P4P and control groups

There were 5478 diabetic patients in the P4P group and 5478 matched controls, with median follow-up periods of 4.37 and 4.27 years, respectively. Table 1 shows the baseline social demographic characteristics, the use of antidiabetic agents and comorbidities. There were no significant differences in age and gender between the 2 groups. Although duration of diabetes and mean follow-up time showed statistically significant by 1-month difference but in terms of clinical effect was small. The frequency of outpatient visits (times/year) was higher in the P4P group than in the control group (11.87 times/year vs 7.4 times/years, *P* < .0001). The control group had more comorbidities than the P4P group (Table 1).

**Table 1:**
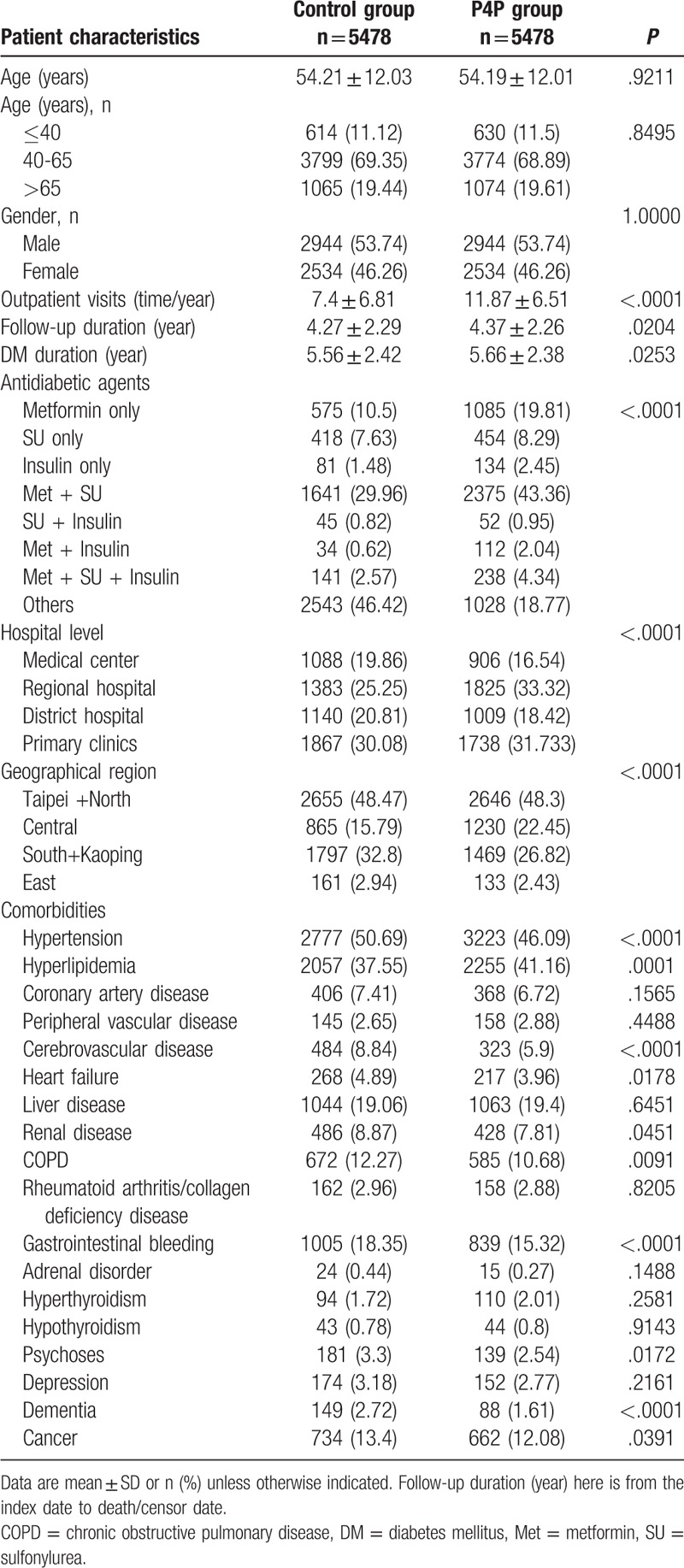
Demographic and clinical characteristics of the P4P and control subjects.

### 3.2 Incidence of mortality

Of the 5478 patients in each group, 250 died in the P4P group and 395 in the control group from 2002 to 2010 (mortality rate 104 vs 169 per 10,000 person-years, respectively, *P* < .0001).

### 3.3 Effect of the P4P program on the incidence of mortality

In the Cox proportional hazards model, the individuals participating in the P4P program had a significantly lower mortality rate after multivariate adjustments (aHR 0.58 [95% CI (0.48–0.69)]) (Table 2). With each 1-year increase in age, the annual mortality rate increased by approximately 5%. Compared with the patients who used metformin only, the aHRs [95% CI] for the use of insulin only, sulfonylurea+insulin, metformin+insulin, and metformin+sulfonylurea+insulin were 6.52 [4.37–9.73], 8.69 [5.45–13.85], 7.56 [4.78–11.98] and 8.49 [5.93–12.15], respectively. Several baseline comorbidities were associated with an increased risk of mortality: cerebrovascular disease (aHR [95% CI]:1.34 [1.07–1.67]), liver disease (aHR [95% CI]:1.24 [1.02–1.52]), adrenal disorders (aHR [95% CI]:2.58 [1.39–4.77]) and psychoses (aHR [95% CI]:1.65 [1.01–2.69]), although hyperlipidemia was associated with a lower risk of mortality (aHR [95% CI]:0.67 [0.56–0.80]).

**Table 2:**
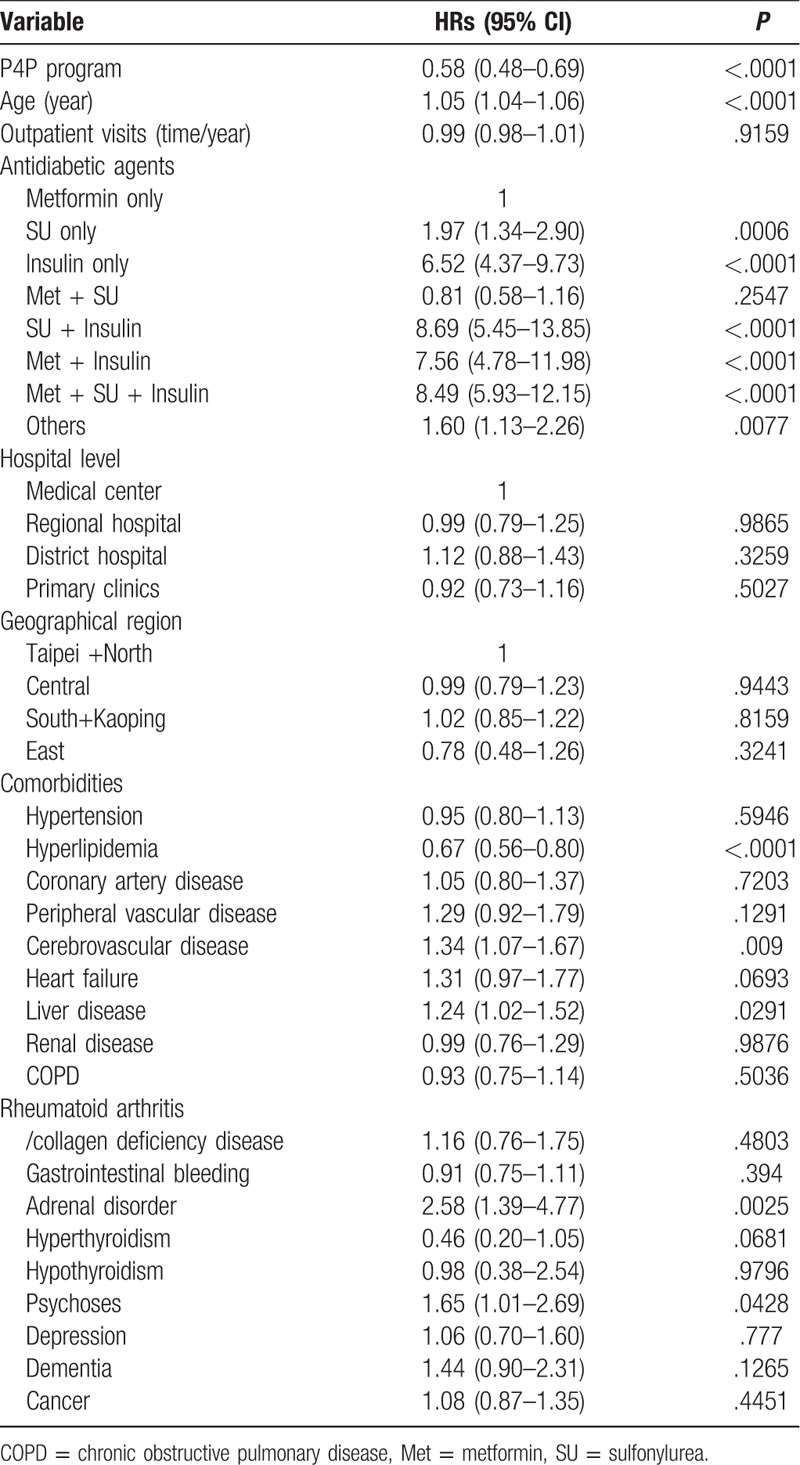
Association between clinical and demographic variables and mortality.

### 3.4 Association between the duration of P4P adherence and mortality

In total, 3267 and 2008 patients achieved good P4P adherence for at least 1 year and at least 2 years, respectively. Compared with their matched controls, the better the adherence to the P4P program, the greater the reduction in mortality rate, with aHRs [95% CI] of 0.48 [0.38–0.62] and 0.36 [0.26–0.49], respectively, in the patients with a minimum of 1 year and 2 years good adherence to the P4P program (Table 3). Regarding the antidiabetic agents, insulin use with or without metformin/sulfonylurea was consistently associated with an increase in mortality in both subgroups. We then investigated whether the patients with good adherence to the P4P program still demonstrated survival benefits compared with those with poor adherence. Among the 5478 enrollees in the P4P program, there were 3470 and 2008 patients in the good adherence and poor adherence groups, respectively, according to the adherence status stratified by achieving good P4P adherence for at least 2 years (Supplemental Table 1). The effect of the reduction in mortality was seen in the good adherence group (0.46 [0.34–0.61]) compared with the poor adherence group after multivariate adjustments (Supplemental Table 2).

**Table 3:**
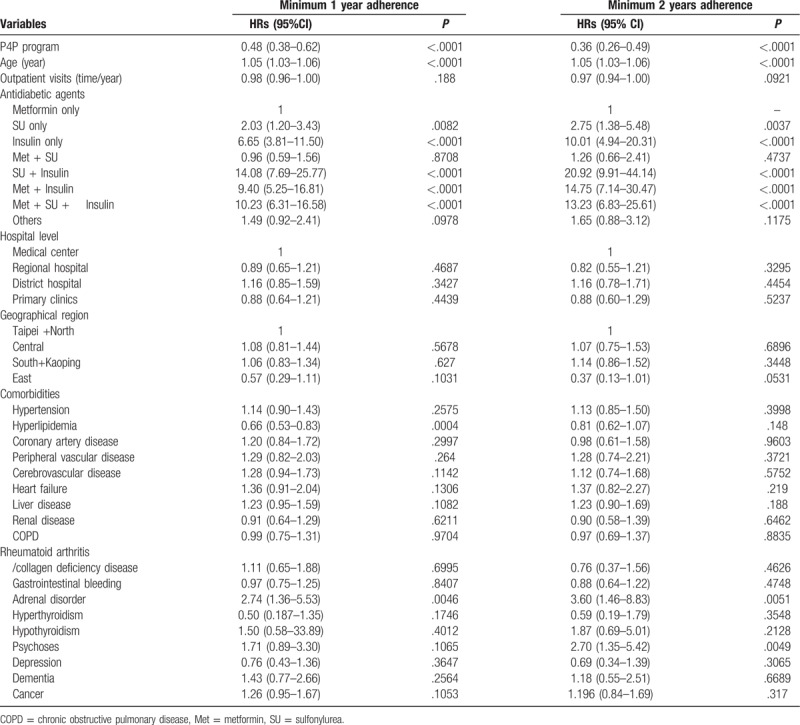
Association between duration of good P4P adherence and mortality.

### 3.5 Multivariate stratified analysis and Kaplan–Meier survival estimates

**Figure 1:**
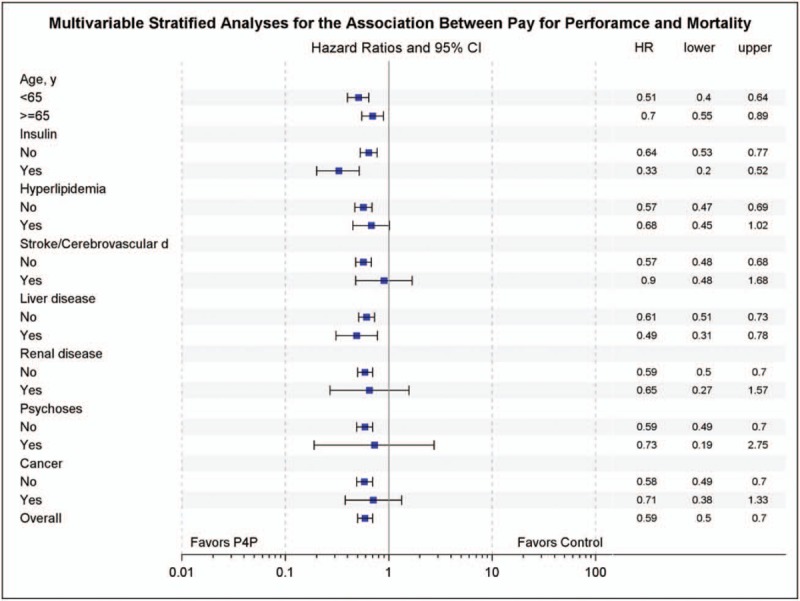
Multivariate stratified analysis for associations between the pay for performance program and mortality.

The P4P cohort was associated with a reduced mortality rate in most of the stratified analysis, including the elderly (0.69 [0.53–0.90]), insulin users (0.47 [0.35–0.63]), liver disease (0.52 [0.33–0.80]), and renal disease (0.40 [0.20–0.78]) (Fig. 1). Kaplan–Meier survival curves showed a significance difference between the P4P group and control group (*P* < .0001 by the log rank test) (Supplemental Figure 1).

## 4 Discussion

The results showed that within 5 years of the diagnosis of type 2 diabetes, participating in the P4P program was associated with a significant reduction in all-cause mortality compared with standard medical care alone. The positive effects of the P4P program existed across age- and disease-specific groups except for those with stroke, cancer and psychosis, which may be explained by high competing mortality of these diseases^[22–24]^ and poor compliance due to the underlying diseases and conditions that can lead to a diminished P4P effect. In the subgroup analysis, the patients with a longer regular adherence to the P4P program had a greater reduction in mortality rate. Hence, both participating in and adhering to the P4P program played an important role in contributing to the reduction of mortality in the patients with newly diagnosed type 2 diabetes.

Previous observational studies in Germany suggested the benefit of a diabetes disease management program in improving patient survival,^[25,26]^ however, Miksch et al^[26]^ excluded patients aged younger 50 years, those with a longer duration of diabetes, and patients enrolled mainly from a single or regional health fund cannot be representative of a national population. The generalizability of the results may be limited due to the selection of the patients in these trials. Recent studies in Taiwan has shown the potential benefit of diabetes P4P programs in reducing risk of all-cause mortality among type 2 diabetic patients,^[12,13]^ however, the enrolled subjects may or may not be newly diagnosed type 2 diabetes mellitus, the number of years since diabetes was diagnosed could not be traced and lack of the time interval between the diagnosis of type 2 diabetes mellitus and enrolled date of P4P program.

A P4P program is a coordinated and proactive multidisciplinary approach with a focus on diet advice, behavior modification, and multifactorial risk reduction. The study showed the positive impact of diabetes self-management behavior, an important element of patient education in a P4P program, on the reduction of all-cause mortality in patients with type 2 diabetes.^[27]^ The Steno-2 study also confirmed that multifactorial risk factor interventions improve morbidity and mortality outcomes in patients with diabetes.^[28,29]^ Moreover, 1 study from the UK found that mean blood pressure and blood glucose were reduced after the introduction of a P4P incentive program for patients with type 2 diabetes^[30]^ and another study from Taiwan has shown that the P4P patients tended to receive more HbA1c tests, fundus and foot examinations, had good HbA1c and LDL outcomes (HbA1c < 7%, LDL < 100 mg/dl) and less likely to have poor HbA1c and LDL outcomes (HbA1c > 9.5%, LDL > 130 mg/dl).^[5]^

However, the optimal timing for when to enroll in a P4P program after the diagnosis of diabetes has not been established. It is possible that the benefits of a legacy effect in vascular protection may not be manifested and the habit of diabetes self-care management not be deeply ingrained if the initiation of a P4P program after diabetes diagnosis occurs too late. Our findings suggest positive survival benefits of a P4P program initiated within 5 years after the diagnosis of diabetes, however, the survival benefits beyond 5 years are unknown. It is possible that a longer duration of diabetes is associated with an increased risk of coronary heart disease^[31]^ and increased prevalence of cancer^[32]^ which may attenuate the effects of a P4P program, and therefore large prospective clinical trials are needed to explore whether survival benefits exist with enrollment in a P4P program beyond 5 years after the diagnosis of diabetes.

It is clear that noncompliance including non-adherence to medication and clinic appointments is linked to worse metabolic status. Adults aged 45 to 64 years, the most common age for a diagnosis of diabetes, have been reported to have poor glycemic control, adverse health-related behavior, and to receive less guideline-recommended examinations than those aged 65 years or older.^[33]^ Currie et al^[34]^ demonstrated that poor medical compliance was associated with an increase in all-cause mortality in patients with type 2 diabetes treated with insulin. Our results showed that patients enrolled in the P4P program made more outpatient visits than those who were not enrolled, consistent with prior studies.^[3,4]^ It is plausible that the patients with poor adherence may have developed more diabetic complications over time which thereby increased the risk of all-cause mortality. Our results revealed that the reduction in mortality was strongly correlated with the duration of adherence to the P4P program, regardless of the treatment modality (insulin or oral antidiabetic agents). Indeed, the major difference between patients with and without participating the P4P program is favored in the diabetes education and adherence to diet, medications and guideline-recommended examinations, rather than the medication they used. Because it is not possible for physicians to prescribe different medications or set different therapeutic targets according to patient participating P4P program or not. In contrast, team members can spend more time for patients in P4P program to educate more detailed knowledge and skills of diabetes care, which might minimize the incidences of diabetic complications. A good interrelationship between members and patients can also be established through this process and can motivate patients to get more adherences to the subsequent medical managements. Thus, interventions to improve adherence to P4P programs should be implemented in clinical practice.

Insulin exposure seemed to be significantly correlated with mortality in our results. Using insulin alone or insulin plus metformin, sulfonylurea, or both resulted in an increased risk of mortality compared with using metformin alone, which suggests that exogenous insulin treatment has a detrimental effect on survival benefits and appears to be of prognostic importance. The majority of previous studies have reported that the administration of exogenous insulin leads to adverse outcomes of diabetes-related complications and all-cause mortality.^[35–38]^ The underlying mechanisms are unknown, however it is plausible that insulin is associated with an increased risk of developing hypoglycemia, cardiovascular disease,^[39,40]^ and cancer^[41,42]^, which in turn increase the risk of sudden death due to hypoglycemia-induced cardiac arrhythmia^[43]^, death from cardiovascular events and cancer.^[44]^ Nevertheless, most previous studies have been observational research, associated with inherent bias and confounding factors so that the results should be interpreted with caution. In real world clinical practice, the patients with using insulin therapy may reflect the more difficult glycemic control, which may infer the underlying more complicated metabolic status and a larger degree of comorbidities. Whether or not insulin has a detrimental effect on mortality remains controversial and particular care should be taken when prescribing insulin.

Our results suggest that participating in a P4P program is associated with survival benefits; however, these results should be interpreted within the context of the studys limitation. First, given that this is an observational study, possible residual confounding factors cannot be excluded. We individually matched pairs for age, gender and the date of a diagnosis of diabetes, and both groups were followed-up from the same enrollment date to avoid immortal time bias,^[45]^ which is common in cohort studies. Although we took comorbidities, social-demographics including the level and location of the medical hospital into account, we were unable to adjust for other aspects of care including personal glycemic control such as HbA1c level, blood pressure control, hyperlipidemia control such as LDL level, educational status, body mass index and physical activity, which are not available from the NHIRD but may have influenced the results. Second, the people who willingly participate in a P4P program or are selected for such disease management programs by physicians are not randomly selected. Many patient- and physician- level factors about participating in P4P program could be contributing to selection bias. Hsieh et al^[46]^ has reported that patients with greater disease severity and comorbidities were more likely to be excluded from the P4P program. As similar phenomenon shown in our study, the non-P4P group had more comorbidities than the P4P group. The enrollees may be more conscious and concerned about their health care than people who do not enroll or drop out early, also known as the “healthy user effect”.^[47]^ However, we cannot account for these biases, which may result in biased estimates of the effect on health outcomes.

## 5 Conclusions

In conclusion, participating in a P4P program within 5 years after the diagnosis of type 2 diabetes was associated with a significant reduction in all-cause mortality independently of underlying comorbidities and other covariates. Moreover, the patients with better adherence had a better survival rate. With the ever increasing number of patients with diabetes worldwide and the associated higher risks of developing cardiovascular disease, chronic kidney disease and cancer in addition to the large economic burden on healthcare systems, we suggest that efforts should be devoted to promoting P4P programs in patients with newly diagnosed type 2 diabetes for long-term survival benefits.

## Author contributions

**Conceptualization:** Fang-Ping Kung.

**Data curation:** Ching-Fang Tsai, Li-Chung Huang.

**Formal analysis:** Fang-Ping Kung, Ching-Fang Tsai, Chin-Li Lu.

**Investigation:** Fang-Ping Kung.

**Methodology:** Ching-Fang Tsai, Chin-Li Lu.

**Project administration:** Fang-Ping Kung.

**Software:** Ching-Fang Tsai, Chin-Li Lu.

**Writing – original draft:** Fang-Ping Kung.

**Writing – review & editing:** Chieh-Hsiang Lu.

## Supplementary Material

Supplemental Digital Content

## Supplementary Material

Supplemental Digital Content

## Supplementary Material

Supplemental Digital Content

## Supplementary Material

Supplemental Digital Content

## References

[1] Gregg EW Zhuo X Cheng YJ Trends in lifetime risk and years of life lost due to diabetes in the USA, 1985-2011: a modelling study. Lancet Diabetes Endocrinol 2014;2:867–74. 25128274 10.1016/S2213-8587(14)70161-5

[2] World Health Organization. 50 Facts: Global health situation and trends 1955–2025, https://www.who.int/whr/1998/media_centre/50facts/en/. 2015. Accessed March 12, 2018.

[3] Lai CL Hou YH The association of clinical guideline adherence and pay-for-performance among patients with diabetes. J Chin Med Assoc 2013;76:102–7. 23351421 10.1016/j.jcma.2012.06.024

[4] Lee TT Cheng SH Chen CC A pay-for-performance program for diabetes care in Taiwan: a preliminary assessment. Am J Manag Care 2010;16:65–9. 20148607

[5] Chiu HC Hsieh HM Lin YC Patient assessment of diabetes care in a pay-for-performance program. Int J Qual Health Care 2016;28:183–90. 26819445 10.1093/intqhc/mzv120

[6] LeBlanc E Belanger M Thibault V Influence of a Pay-for-Performance Program on Glycemic Control in Patients Living with Diabetes by Family Physicians in a Canadian Province. Can J Diabetes 2017;41:190–6. 27908559 10.1016/j.jcjd.2016.09.008

[7] Sidorov J Shull R Tomcavage J Does diabetes disease management save money and improve outcomes? A report of simultaneous short-term savings and quality improvement associated with a health maintenance organization-sponsored disease management program among patients fulfilling health employer data and information set criteria. Diabetes Care 2002;25:684–9. 11919125 10.2337/diacare.25.4.684

[8] Yu HC Tsai WC Kung PT Does the pay-for-performance programme reduce the emergency department visits for hypoglycaemia in type 2 diabetic patients? Health Policy Plan 2014;29:732–41. 23894069 10.1093/heapol/czt056

[9] Guzder RN Gatling W Mullee MA Early mortality from the time of diagnosis of Type 2 diabetes: a 5-year prospective cohort study with a local age- and sex-matched comparison cohort. Diabet Med 2007;24:1164–7. 17672858 10.1111/j.1464-5491.2007.02223.x

[10] Nwaneri C Cooper H Jones DB Mortality in type 2 diabetes mellitus- magnitude of the evidence from a systematic review and meta-analysis. Br J Diabetes Vasc Dis 2013;13:192–207.

[11] Holman RR Paul SK Bethel MA 10-year follow-up of intensive glucose control in type 2 diabetes. N Engl J Med 2008;359:1577–89. 18784090 10.1056/NEJMoa0806470

[12] Hsieh HM He JS Shin SJ A diabetes pay-for-performance program and risks of cancer incidence and death in patients with type 2 diabetes in Taiwan. Prev Chronic Dis 2017;14:E88. 28981404 10.5888/pcd14.170012 PMC5645199

[13] Chen YC Lee CT Lin BJ Impact of pay-for-performance on mortality in diabetes patients in Taiwan: A population-based study. Medicine (Baltimore) 2016;95:e4197. 27399144 10.1097/MD.0000000000004197 PMC5058873

[14] Hsieh CY Su CC Shao SC Taiwan's National Health Insurance Research Database: past and future. Clin Epidemiol 2019;11:349–58. 31118821 10.2147/CLEP.S196293 PMC6509937

[15] Hanberg JS Tang WHW Wilson FP An exploratory analysis of the competing effects of aggressive decongestion and high-dose loop diuretic therapy in the DOSE trial. Int J Cardiol 2017;241:277–82. 28392080 10.1016/j.ijcard.2017.03.114 PMC5471358

[16] Hsieh CY Chen CH Li CY Validating the diagnosis of acute ischemic stroke in a National Health Insurance claims database. J Formos Med Assoc 2015;114:254–9. 24140108 10.1016/j.jfma.2013.09.009

[17] Cheng CL Kao YH Lin SJ Validation of the National Health Insurance Research Database with ischemic stroke cases in Taiwan. Pharmacoepidemiol Drug Saf 2011;20:236–42. 21351304 10.1002/pds.2087

[18] Hsieh CY Cheng CL Lai EC Identifying renal dysfunction in stroke patients using diagnostic codes in the Taiwan National Health Insurance Research Database. Int J Stroke 2015;10:E5. 25491551 10.1111/ijs.12380

[19] Kao WH Hong JH See LC Validity of cancer diagnosis in the National Health Insurance database compared with the linked National Cancer Registry in Taiwan. Pharmacoepidemiol Drug Saf 2018;27:1060–6. 28815803 10.1002/pds.4267

[20] Dekker FW de Mutsert R van Dijk PC Survival analysis: time-dependent effects and time-varying risk factors. Kidney Int 2008;74:994–7. 18633346 10.1038/ki.2008.328

[21] Wolfe RA Strawderman RL Logical and statistical fallacies in the use of Cox regression models. Am J Kidney Dis 1996;27:124–9. 8546126 10.1016/s0272-6386(96)90039-6

[22] Ranc K Jorgensen ME Friis S Mortality after cancer among patients with diabetes mellitus: effect of diabetes duration and treatment. Diabetologia 2014;57:927–34. 24633676 10.1007/s00125-014-3186-z

[23] Tuomilehto J Rastenyte D Jousilahti P Diabetes mellitus as a risk factor for death from stroke. Prospective study of the middle-aged Finnish population. Stroke 1996;27:210–5. 8571411 10.1161/01.str.27.2.210

[24] De Hert M Dekker JM Wood D Cardiovascular disease and diabetes in people with severe mental illness position statement from the European Psychiatric Association (EPA), supported by the European Association for the Study of Diabetes (EASD) and the European Society of Cardiology (ESC). Eur Psychiatry 2009;24:412–24. 19682863 10.1016/j.eurpsy.2009.01.005

[25] Stock S Drabik A Buscher G German diabetes management programs improve quality of care and curb costs. Health Aff (Millwood) 2010;29:2197–205. 21134920 10.1377/hlthaff.2009.0799

[26] Miksch A Laux G Ose D Is there a survival benefit within a German primary care-based disease management program? Am J Manag Care 2010;16:49–54. 20148605

[27] Laxy M Mielck A Hunger M The association between patient-reported self-management behavior, intermediate clinical outcomes, and mortality in patients with type 2 diabetes: results from the KORA-A study. Diabetes Care 2014;37:1604–12. 24667462 10.2337/dc13-2533

[28] Gaede P Lund-Andersen H Parving HH Effect of a multifactorial intervention on mortality in type 2 diabetes. N Engl J Med 2008;358:580–91. 18256393 10.1056/NEJMoa0706245

[29] Gaede P Vedel P Larsen N Multifactorial intervention and cardiovascular disease in patients with type 2 diabetes. N Engl J Med 2003;348:383–93. 12556541 10.1056/NEJMoa021778

[30] Millett C Netuveli G Saxena S Impact of pay for performance on ethnic disparities in intermediate outcomes for diabetes: a longitudinal study. Diabetes Care 2009;32:404–9. 19073759 10.2337/dc08-0912 PMC2646017

[31] Fox CS Sullivan L D’Agostino RB Sr The significant effect of diabetes duration on coronary heart disease mortality: the Framingham Heart Study. Diabetes Care 2004;27:704–8. 14988289 10.2337/diacare.27.3.704

[32] Li C Zhao G Okoro CA Prevalence of diagnosed cancer according to duration of diagnosed diabetes and current insulin use among U.S. adults with diagnosed diabetes: findings from the 2009 Behavioral Risk Factor Surveillance System. Diabetes Care 2013;36:1569–76. 23300288 10.2337/dc12-1432 PMC3661832

[33] O’Connor PJ Desai JR Solberg LI Variation in diabetes care by age: opportunities for customization of care. BMC Fam Pract 2003;4:16. 14585101 10.1186/1471-2296-4-16 PMC280680

[34] Currie CJ Peyrot M Morgan CL The impact of treatment noncompliance on mortality in people with type 2 diabetes. Diabetes Care 2012;35:1279–84. 22511257 10.2337/dc11-1277 PMC3357221

[35] Currie CJ Poole CD Evans M Mortality and other important diabetes-related outcomes with insulin vs other antihyperglycemic therapies in type 2 diabetes. J Clin Endocrinol Metab 2013;98:668–77. 23372169 10.1210/jc.2012-3042 PMC3612791

[36] Gamble JM Simpson SH Eurich DT Insulin use and increased risk of mortality in type 2 diabetes: a cohort study. Diabetes Obes Metab 2010;12:47–53. 19788429 10.1111/j.1463-1326.2009.01125.x

[37] Roumie CL Greevy RA Grijalva CG Association between intensification of metformin treatment with insulin vs sulfonylureas and cardiovascular events and all-cause mortality among patients with diabetes. JAMA 2014;311:2288–96. 24915260 10.1001/jama.2014.4312 PMC4149288

[38] Mellbin LG Malmberg K Norhammar A Prognostic implications of glucose-lowering treatment in patients with acute myocardial infarction and diabetes: experiences from an extended follow-up of the Diabetes Mellitus Insulin-Glucose Infusion in Acute Myocardial Infarction (DIGAMI) 2 Study. Diabetologia 2011;54:1308–17. 21359582 10.1007/s00125-011-2084-x

[39] Margolis DJ Hoffstad O Strom BL Association between serious ischemic cardiac outcomes and medications used to treat diabetes. Pharmacoepidemiol Drug Saf 2008;17:753–9. 18613215 10.1002/pds.1630 PMC2635115

[40] Stout RW Hyperinsulinemia and atherosclerosis. Diabetes 1996;45: Suppl 3: S45–6. 8674889 10.2337/diab.45.3.s45

[41] Yang YX Hennessy S Lewis JD Insulin therapy and colorectal cancer risk among type 2 diabetes mellitus patients. Gastroenterology 2004;127:1044–50. 15480982 10.1053/j.gastro.2004.07.011

[42] Currie CJ Poole CD Gale EA The influence of glucose-lowering therapies on cancer risk in type 2 diabetes. Diabetologia 2009;52:1766–77. 19572116 10.1007/s00125-009-1440-6

[43] Nordin C The case for hypoglycaemia as a proarrhythmic event: basic and clinical evidence. Diabetologia 2010;53:1552–61. 20407743 10.1007/s00125-010-1752-6

[44] Bowker SL Yasui Y Veugelers P Glucose-lowering agents and cancer mortality rates in type 2 diabetes: assessing effects of time-varying exposure. Diabetologia 2010;53:1631–7. 20407744 10.1007/s00125-010-1750-8

[45] Levesque LE Hanley JA Kezouh A Problem of immortal time bias in cohort studies: example using statins for preventing progression of diabetes. BMJ 2010;340:b5087. 20228141 10.1136/bmj.b5087

[46] Hsieh HM Tsai SL Mau LW Effects of changes in diabetes pay-for-performance incentive designs on patient risk selection. Health Serv Res 2016;51:667–86. 26152649 10.1111/1475-6773.12338 PMC4799909

[47] Shrank WH Patrick AR Brookhart MA Healthy user and related biases in observational studies of preventive interventions: a primer for physicians. J Gen Intern Med 2011;26:546–50. 21203857 10.1007/s11606-010-1609-1 PMC3077477

