# Supplementary figures and images for: Diabetes pay-for-performance program can reduce all-cause mortality in patients with newly diagnosed type 2 diabetes mellitus
Source: Medicine (Baltimore). 2020 Feb 14;99(7):e19139. doi: 10.1097/MD.0000000000019139 (PMC7035087; doi:10.1097/MD.0000000000019139)

Supplemental Figure 1. Kaplan-Meier curves for 10-year survival rate


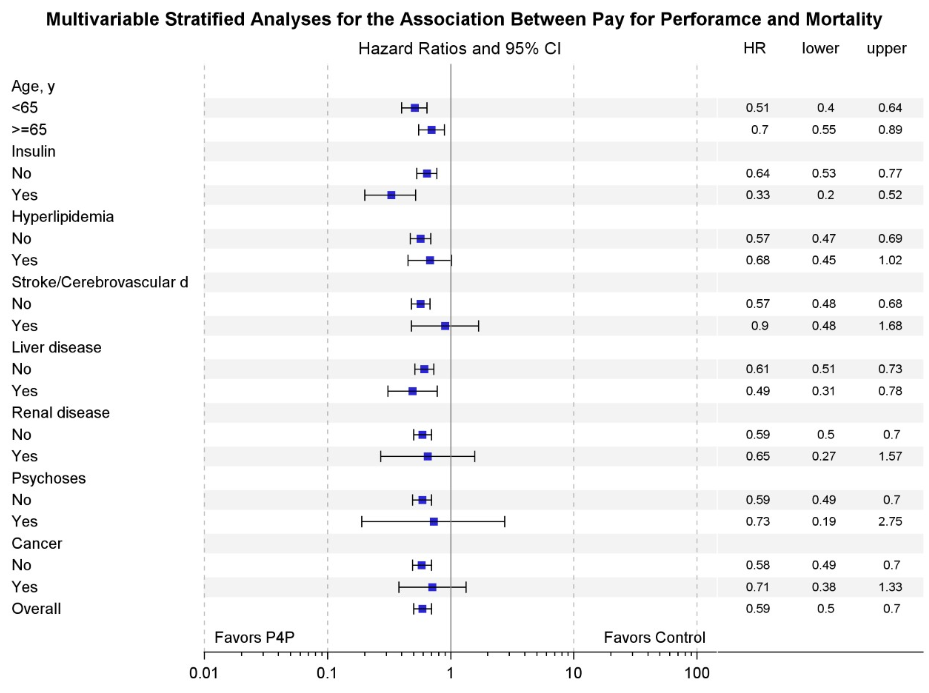

Supplement: Supplemental Digital Content [file medi-99-e19139-s001.doc]

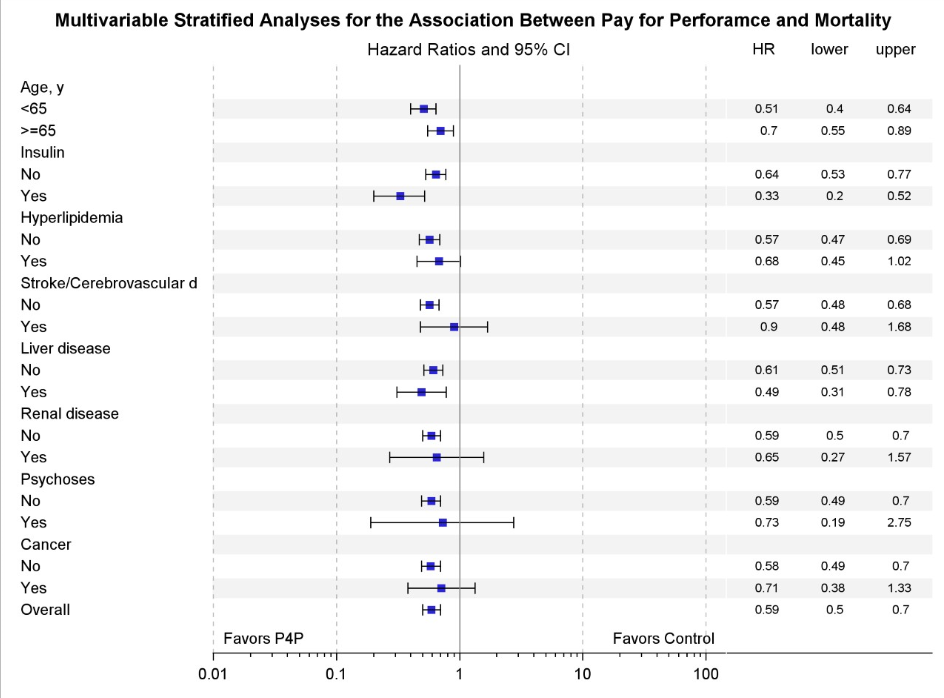

Supplement: Supplemental Digital Content [file medi-99-e19139-s002.doc]
