# Supplementary material for: Diabetes pay-for-performance program can reduce all-cause mortality in patients with newly diagnosed type 2 diabetes mellitus
Source: Medicine (Baltimore). 2020 Feb 14;99(7):e19139. doi: 10.1097/MD.0000000000019139 (PMC7035087; doi:10.1097/MD.0000000000019139)
Supplement: Supplemental Digital Content [file medi-99-e19139-s003.doc]

**Supplemental** Table 1 Demographics and characteristics of P4P population stratified by achieving regular P4P adherence for at least 2 years. (N=5478)

|  | **Good adherence group** | **Poor adherence group** |  |
| --- | --- | --- | --- |
| **Patient characteristics** | **n =3470** | **n =2008** | ***p*** |
| Age (years) | 53.98±12.26 | 54.54±11.56 | 0.0914 |
| Age (years), n |  |  |  |
| ≤40 | 423 (12.19) | 630 (11.5) | 0.0966 |
| 40-65 | 2365 (68.16) | 1409 (70.17) |  |
| >65 | 682 (19.65) | 392 (19.52) |  |
| Gender, n |  |  | 0.0028 |
| Male | 1918 (55.27) | 1026 (51.1) |  |
| Female | 1552 (44.73) | 982 (48.9) |  |
| Follow-up duration (year) | 3.92±2.38 | 5.15±1.8 | <0.0001 |
| DM duration (year) | 5.22±2.47 | 6.442±2.00 | <0.0001 |
| Interval time^*^ | 1.3±1.44 | 1.27±1.39 | 0.438 |
| Outpatient visits (time/year) | 10.98±6.72 | 13.39±5.82 | <0.0001 |
| Antidiabetic agents |  |  |  |
| Metformin only | 652 (18.79) | 428 (21.31) | <0.0001 |
| SU only | 257 (7.41) | 200 (9.96) |  |
| Insulin only | 64 (1.84) | 64 (3.19) |  |
| Met + SU | 1442 (41.56) | 946 (47.11) |  |
| SU + Insulin | 34 (0.98) | 16 (0.8) |  |
| Met + Insulin | 62 (1.79) | 51 (2.54) |  |
| Met + SU + Insulin | 154 (4.44) | 93 (4.63) |  |
| Others | 805 (23.2) | 210 (10.46) |  |
| Hospital level |  |  | <0.0001 |
| Medical center | 642 (18.5) | 264 (13.15) |  |
| Regional hospital | 1186 (34.18) | 639 (31.82) |  |
| District hospital | 598 (17.23) | 411 (20.47) |  |
| Primary clinics | 1044 (30.09) | 694 (34.56) |  |
| Geographical region |  |  | 0.0784 |
| Taipei +North | 1656 (47.72) | 990 (49.3) |  |
| Central | 758 (21.84) | 472 (23.51) |  |
| South+Kaoping | 969 (27.93) | 500 (24.9) |  |
| East | 87 (2.51) | 46 (2.29) |  |
| Comorbidities |  |  |  |
| Hypertension | 1581(45.56) | 944 (47.01) | 0.2995 |
| Hyperlipidemia | 1410 (40.63) | 845 (42.08) | 0.2941 |
| Coronary artery disease | 225(6.48) | 143 (7.12) | 0.3639 |
| Peripheral vascular disease | 105 (3.03) | 53 (2.64) | 0.4102 |
| Cerebrovascular disease | 191 (5.5) | 132 (6.57) | 0.1054 |
| Heart failure | 147 (4.24) | 70 (3.49) | 0.1701 |
| Liver disease | 657(18.93) | 406 (20.22) | 0.2464 |
| Renal disease | 264 (7.61) | 164 (8.17) | 0.4574 |
| COPD | 353(10.17) | 232 (11.55) | 0.1108 |
| Rheumatoid arthritis/collagen deficiency disease | 96 (2.77) | 62 (3.09) | 0.4938 |
| Gastrointestinal bleeding | 528 (15.22) | 311 (15.49) | 0.7877 |
| Adrenal disorder | 9 (0.2) | 6 (0.3) | 0.7878 |
| Hyperthyroidism | 60 (1.73) | 50 (2.49) | 0.053 |
| Hypothyroidism | 23 (0.66) | 21 (1.05) | 0.126 |
| Psychoses | 88 (2.54) | 61 (3.04) | 0.9931 |
| Depression | 91 (2.62) | 152 (2.77) | 0.3671 |
| Dementia | 59 (1.7) | 29 (1.44) | 0.4676 |
| Cancer | 423 (12.19) | 239 (11.9) | 0.7528 |

^*^ Interval time: time between the diagnosis of diabetes mellitus (DM) and the index date. SU, sulfonylurea. Met, metformin. COPD, chronic obstructive pulmonary disease.
