# Supplementary material for: Diabetes pay-for-performance program can reduce all-cause mortality in patients with newly diagnosed type 2 diabetes mellitus
Source: Medicine (Baltimore). 2020 Feb 14;99(7):e19139. doi: 10.1097/MD.0000000000019139 (PMC7035087; doi:10.1097/MD.0000000000019139)
Supplement: Supplemental Digital Content [file medi-99-e19139-s004.doc]

**Supplemental Table 2 Association between P4P adherence and mortality**

| **Variable** | **HRs (95% CI)** | ***p*** |
| --- | --- | --- |
| Good P4P adherence | 0.462 (0.34-0.61) | <0.0001 |
| Age ( per-1 year ) | 1.05 (1.04-1.06) | <0.0001 |
| Male | 1.88 (1.43-2.46) | <0.0001 |
| Interval time^*^ (per-1 year) | 1.01 (0.92-1.12) | 0.7269 |
| Antidiabetic agents |  |  |
| Metformin only | 1 |  |
| SU only | 1.77 (0.99-3.15) | 0.0504 |
| Insulin only | 3.67 (1.87-7.18) | 0.0001 |
| Met + SU | 0.71 (0.42-1.18) | 0.1942 |
| SU + Insulin | 5.12 (2.50-10.48) | <0.0001 |
| Met + Insulin | 8.20 (4.54-14.80) | <0.0001 |
| Met + SU + Insulin | 8.56 (5.20-14.09) | <0.0001 |
| Others | 1.51 (0.92-2.47) | 0.0997 |
| Hospital level |  |  |
| Medical center | 1 |  |
| Regional hospital | 1.47 (0.96-2.26) | 0.0755 |
| District hospital | 1.49 (0.94-2.36) | 0.0873 |
| Primary clinics | 1.64 (1.05-2.55) | 0.0273 |
| Geographical region |  |  |
| Taipei +North | 1 |  |
| Central | 0.92 (0.66-1.26) | 0.60 |
| South+Kaoping | 0.76 (0.55-1.06) | 0.1164 |
| East | 0.40 (0.12-1.28) | 0.1245 |
| Comorbidities |  |  |
| Hypertension | 1.24 (0.93-1.65) | 0.1287 |
| Hyperlipidemia | 0.82 (0.61-1.09) | 0.1862 |
| Coronary artery disease | 1.01 (0.64-1.57) | 0.9615 |
| Peripheral vascular disease | 1.07 (0.60-1.89) | 0.8158 |
| Cerebrovascular disease | 1.36 (0.93-2.01) | 0.1097 |
| Heart failure | 0.83 (0.48-1.43) | 0.5143 |
| Liver disease | 1.16 (0.84-1.59) | 0.3559 |
| Renal disease | 0.83 (0.53-1.29) | 0.4264 |
| COPD | 1.10 (0.77-1.56) | 0.5784 |
| Rheumatoid arthritis  /collagen deficiency disease | 1.52 (0.79-2.94) | 0.2052 |
| Gastrointestinal bleeding | 0.88 (0.64-1.23) | 0.4758 |
| Adrenal disorder | 3.12 (0.73-13.33) | 0.124 |
| Hyperthyroidism | 1.24 (0.45-3.41) | 0.6775 |
| Hypothyroidism | 0.49 (0.06-3.66) | 0.4907 |
| Psychoses | 1.71 (0.80-3.65) | 0.1648 |
| Depression | 1.14 (0.60-2.18) | 0.6761 |
| Dementia | 1.96 (0.93-4.16) | 0.0759 |
| Cancer | 1.27 (0.89-1.79) | 0.1759 |

^*^ Interval time: time between the diagnosis of diabetes mellitus (DM) and the index date. SU, sulfonylurea. Met, metformin. COPD, chronic obstructive pulmonary disease.
